# Comparison of anti-cancer effects of novel protein disulphide isomerase (PDI) inhibitors in breast cancer cells characterized by high and low PDIA17 expression

**DOI:** 10.1186/s12935-022-02631-w

**Published:** 2022-06-20

**Authors:** Anna Kurpińska, Joanna Suraj-Prażmowska, Marta Stojak, Joanna Jarosz, Łukasz Mateuszuk, Ewa Niedzielska-Andres, Magdalena Smolik, Joanna Wietrzyk, Ivars Kalvins, Maria Walczak, Stefan Chłopicki

**Affiliations:** 1 Jagiellonian Centre for Experimental Therapeutics (JCET), Jagiellonian University, Bobrzynskiego 14, 30-348 Krakow, Poland; 2 Hirszfeld Institute of Immunology and Experimental Therapy, Department of Experimental Oncology, Polish Academy of Sciences, Rudolfa Weigla 12, 53-114 Wroclaw, Poland; 3 Faculty of Pharmacy, Chair and Department of Toxicology, Jagiellonian University Medical College, Medyczna 9, 30-688 Krakow, Poland; 4 Laboratory of Carbofunctional Compounds, Latvian Institute of Organic Synthesis, Riga, 1006 Latvia; 5 Faculty of Medicine, Chair of Pharmacology, Jagiellonian University Medical College, Grzegorzecka 16, 31-531 Krakow, Poland

**Keywords:** Protein disulphide isomerases, Anterior gradient 2 (AGR2, PDIA17), Novel PDI inhibitors, Endoplasmic reticulum (ER)–resident protein 57 (ERP57, PDIA3), Protein disulphide isomerase A1 (prolyl 4-hydroxylase subunit beta, P4HB, PDIA1), Cancer cell lines

## Abstract

**Background:**

Protein disulphide isomerases (PDIs) play an important role in cancer progression. However, the relative contribution of the various isoforms of PDI in tumorigenesis is not clear.

**Methods:**

The content of PDI isoforms in 22 cancer cells lines was investigated using LC–MS/MS-based proteomic analysis. The effects of PDIA1, PDIA3 and PDIA17 inhibition on the proliferation, migration and adhesion of MCF-7 and MDA-MB-231 cells, identified as high and low PDIA17 expressing cells, respectively, were assessed using novel aromatic N-sulphonamides of aziridine-2-carboxylic acid derivatives as PDI inhibitors.

**Results:**

PDIA1 and PDIA3 were the most abundant in cancer cell lysates and were also detected extracellularly in breast cancer cells (MDA-MB-231 and MCF-7). Some cancer cell lines (e.g., MCF-7, HT-29) showed upregulated expression of PDIA17, whereas in others (e.g., MDA-MB-231, 67NR), PDIA17 was not detected. The simultaneous inhibition of PDIA1 and PDIA3 showed similar anti-proliferative effects in MCF-7 and MDA-MB-231 breast cancer cells. However, the inhibition of PDIA1 and PDIA17 in the MCF-7 cell line resulted in more effective anti-adhesive and anti-proliferative effects.

**Conclusions:**

PDIA1 and PDIA3 represent major isoforms of multiple cancer cells, and their non-selective inhibition displays significant anti-proliferative effects irrespective of whether or not PDIA17 is present. The more pronounced anti-adhesive effects of PDI inhibition in hormone-sensitive MCF-7 cells featured by higher levels of PDIs when compared to triple-negative MDA-MB-231 cells suggests that targeting extracellular PDIA1 and PDIA3 with or without additional PDIA17 inhibition may represent a strategy for personalized anti-adhesive, anti-metastatic therapy in cancers with high PDI expression.

**Supplementary Information:**

The online version contains supplementary material available at 10.1186/s12935-022-02631-w.

## Background

Protein disulphide isomerases (PDIs) are highly expressed proteins present predominantly in the endoplasmic reticulum but also in the cytosol, nucleus, on the cell surface and extracellularly [1]. PDIs were initially identified as chaperone proteins and are involved in the reduction, oxidation and isomerization of disulphide bonds [2]. Various functions of PDIs have been proposed, including the regulation of cancer cell phenotype. Indeed, the overexpression of PDIs in highly metabolically active cancer cells maintains the proper folding of the proteins and prevents protein aggregation [3].

The best described PDI isoforms in cancer cells are PDIA1 (P4HB) and PDIA3 (ERP57) [4–9]. Suppression of cancer growth by PDIA1 inhibition was proposed as a novel approach to target cancer. Some PDI inhibitors (e.g., quercetin-3 rutinoside and PACMA 31) demonstrate promising anti-cancer activity [10]. Recently, it was shown that extracellular PDIA1 plays an important role in regulating the adhesion of cancer cells to the endothelium and their transendothelial migration, whereas intracellular PDIA1 was involved in regulating cell cycle and caspase 3/7 activation [11]. Additionally, growing evidence supports the important role of PDIA17 (anterior gradient protein 2, AGR2) in oncoproteins, stimulating proliferation and promoting metastasis [4, 12]. Accordingly, among the PDI proteins covering over 20 isoforms, PDIA17 has also emerged as a novel prometastatic and proangiogenic protein and an attractive target for anti-cancer therapy [13, 14].

It is still inconclusive which PDI isoform should be targeted in anti-cancer treatment and whether targeting single rather than a formulation of ‘PDI-pan inhibition’ is a better strategy [15–18]. It is also not clear whether the pattern of PDI isoform expression is heterogeneous or homogeneous in various cancer cells. Here, we address this issue by characterizing the PDI repertoire of multiple cancer cell lines to determine which PDI isoforms are the most abundant and that might represent an optimal target for anti-cancer therapy. Then, we studied the effects of inhibiting the most abundant isoforms, including PDIA1 and PDIA3 as well PDIA17 in cancer cells identified as high and low PDIA17 expressing breast cancer cells. As pharmacological tools to inhibit PDIA1, PDIA3 or PDIA17, we used recently developed aromatic N-sulphonamides of aziridine-2-carboxylic acid derivatives [19, 20].

## Methods

### Cell lines

The studies were performed using 22 human and mouse cancer cell lines, representative for breast (MDA-MB-231, 67NR, MCF-7, 4T1, T47D), colon (LOVO, HT-29, CaCo2, 5637), urinary bladder (5637), prostate (LNCaP, PC-3, Du-145, TRAMP C2, TRAMP C1), lung (LLC, NCI-H1703, A549, A427, NCI-H358, NCI-H1299) and ovarian (A2780) cancer as well as two normal cell lines (MCF10A–human mammary epithelial cell line and BALB/3T3 clone 31–regular mouse fibroblast cell line). The type of culture medium, cell culture conditions and number of passages are shown in Additional file 2: Table S1. Cells were maintained in T75 culture flasks at 37 °C in a humidified atmosphere (5% CO_2_). Cells were regularly tested for mycoplasma contamination using the MycoAlert mycoplasma detection kit (Lonza, Basel, Switzerland). Cells in two to five passages were seeded in equal amounts and grown to 90–95% confluence. The medium from cell cultures was collected and clarified from debris by centrifugation (200*g*, 5 min, at room temperature (RT)). The cells were detached using Acutase solution (Sigma-Aldrich, Saint Louis, USA) and washed twice with Dulbecco's phosphate-buffered saline (DPBS; Gibco, Scotland, UK) and centrifuged at 200*g* for 5 min. at RT. The material was frozen at − 80 °C until analysis.

### Preparation of cell lysate and medium for proteomic analyses

Cells were lysed in lysis buffer (2.8 µl/mg sample, 4 °C) containing 7 M urea (Bio-Shop, Burlington, Canada), 2 M thiourea (Bio-Shop, Burlington, Canada), mass spectrometry safe protease and phosphatase inhibitor cocktail (PIC, 1:100) (Sigma-Aldrich, Saint Louis, USA) and 30 mM Tris-HCl, pH 8.0 (Bio-Shop, Burlington, Canada), sonicated on ice and centrifuged (16,000*g*, 15 min., 4 °C), and the supernatant was collected [21].

The medium was lyophilized, resuspended in 1 ml of MilliQ water, and 10 mg protein was processed using a ProteoMiner Protein Enrichment Kit (Bio-Rad, Hercules, USA) according to the procedure of Boschetti and Righetti [22] (see Additional file 1: Methods for more details). For further proteomic analysis, the obtained eluent was incubated overnight with four volumes of ice-cold acetone. After centrifugation (15,000*g*, 30 min., 0 °C), the pellet was dissolved in 50 mM ammonium bicarbonate (ABC) (Sigma-Aldrich, Saint Louis, USA).

The protein concentration in the cell supernatant and the medium processed using the ProteoMiner Protein Enrichment Kit was each time assessed using a Bradford assay (Bio-Rad, Hercules, USA).

### Semi-quantitative proteomic analysis of PDI content using mass spectrometry

The cell lysates and cell medium for proteomic analysis of PDIs were prepared according to a procedure provided by Sitek et al. [21] with slight modifications.

The proteomic analysis of cell lysates was conducted using Dionex UltiMate™ 3000 RSLC System (Thermo Scientific, San Jose, USA) coupled to LTQ XL hybrid ion trap-Orbitrap Discovery mass spectrometer (Thermo Scientific, San Jose, USA) as described by Kurpinska et al. [23].

For the detection and semi-quantitation of PDI isoforms in cell lysates, the exponentially modified protein abundance index (emPAI) calculation was performed using an in-built tool of the Mascot search engine based on protein coverage by the peptide matches in a database search result [24, 25]. The data for PDI semi-quantitation in cell lysates are presented as mean ± SEM.

The repertoire of released PDIs was characterized in the two lines selected for further studies. To define the PDIs secreted by MDA-MB-231 (negative/low PDIA17 expression) and MCF-7 (high PDIA17 expression), cell media were subjected to enhanced proteomic analysis in the Mass Spectrometry Laboratory at the Institute of Biochemistry and Biophysics, Polish Academy of Sciences, Warsaw, Poland [11, 26]. The data for PDIs present in the medium are presented as the average MS signal response of the three most intense tryptic peptides with SEM [27].

Detailed procedures for the proteomic studies are described in Additional file 1: Methods.

### Confirmation of PDIA17 presence or absence in cell lysates using Western blots

Lysates of all cell lines used in the study were checked for PDIA17 presence using Western blotting (see Additional file 1: Methods for details).

### Immunocytochemistry of PDIA17 in MDA-MB-231 and MCF-7 cell lines

The cells MCF-7 and MDA-MB-231 were stained using immunocytochemistry for PDIA17. Complete information on the general cell culture conditions is provided in “Cell lines” section**.** (see Additional file 1: Methods for details).

### Synthesis of aromatic N-sulphonamides of aziridine-2-carboxylic acid derivatives, PDIA1, PDIA3 and PDIA17 inhibitors

Details on the structure and synthesis of novel PDIA1 and PDIA3 inhibitors (C-3380, C-3389, C-3399) and C-3353 that also displayed PDIA17 inhibition properties were described previously [19, 20]. Detailed procedures are described in Additional file 1: Methods.

The PDI inhibitor activity towards PDIA1, PDIA3 and PDIA17 was assessed as an increase in disulphide bond reduction in human insulin in the presence of DL-dithiotreitol (DTT) causing aggregation of its β-chain, analyzed by turbidimetry as presented earlier by Kalvins et al. [19] and Chlopicki et al. [20] (see Additional file 1: Methods for more details). The data presented by Kalvins et al. [19] and Chlopicki et al. [20] on PDIA1 and PDIA3 inhibition showed that C-3380, C-3389 and C-3399 inhibited PDIA1 and PDIA3 in a low micromolar range, whereas C-3353 displayed an inhibitory effect on PDIA1 but not on PDIA3. Additional studies on the potential of C-3380, C-3389 and C-3399 to inhibit PDIA17 revealed that the tested compounds were not effective against PDIA17, whereas C-3353 inhibited PDIA17 at the level of 20.1 µM (IC_50_) (Additional file 3: Table S2).

### Assessment of anti-cancer effects of PDI inhibitors

#### MTT cell viability assay–half-maximal inhibitory concentration (IC_50_) assessment

The proliferation rate of the cells was measured using an MTT assay. (see Additional file 1: Methods for details). IC_50_ parameter was determined as previously described [28].

#### Cancer cell adhesion to collagen type I

The ability of breast cancer cells to adhere to collagen type I after incubation with the selected PDI inhibitors (C-3353, C-3380, C-3389, C-3399) was analyzed using a functional adhesion assay according to the procedure reported by Stojak et al. [11] with minor modifications. Detailed procedures for the cancer cell adhesion studies are described in Additional file 1: Methods.

#### Electric cell-substrate impedance-sensing assays (ECIS)

The migration of MCF-7 and MDA-MB-231 cells was monitored using real-time quantitative wound healing assays and 96W1E + ECIS arrays (Applied Biophysics, Troy, NY, USA) according to the procedure reported by Stojak et al. [11] with minor modifications and C-3353 inhibitor as a tested compound. Detailed procedures for the ECIS studies are described in Additional file 1: Methods.

## Results

### Characteristics of PDI isoform profiles in various cancer cell lines and identification of cell lines with high and low expression of PDIA17

The PDI repertoire detected by LC–MS/MS-based label-free semi-quantitative proteomic analysis in 22 cancer cell line lysates to some extent was specific to the cell type (Table 1) and included the following PDI isoforms: PDIA1 (P4HB), PDIA3 (ERp57), PDIA4 (ERp72), PDIA6 (ERp5), PDIA9 (ERp29), PDIA10 (ERp44), PDIA15 (TXNDC5), PDIA16 (TXNDC12), PDIA 17 (PDIA17) and PDIA18 (ARG3). The total amount of all PDIs was relatively higher in HT-29 and T47D cell lines (PDIs total emPAI%–2.06% and 2.05%, respectively) as compared to 67NR, LLC, NCIH1299 (PDIs total emPAI%–0.58%, 0.43% and 0.40%, respectively). Independent of the total expression level of all PDI isoforms, the most abundant among all detected PDI isoforms were PDIA1 and PDIA3 in most studied cell lines. In being selected for further studies on breast cancer cell lines, PDIA1 and PDIA3 were also present extracellularly (MDA-MB-231 and MCF-7) (Table 2).

**Table 1:** The relative amount of PDI isoforms (PDIome) in selected cancer cell lines

No	Cell line	PDI repertoire in cell lysates	Type of cancer	PDIA1	PDIA3	PDIA4	PDIA6	PDIA9	PDIA10	PDIA15	PDIA16	PDIA17	PDIA18
1	LNCaP	1,3,4,6,9,10	Prostate cancer	0.14 ± 0.03	0.35 ± 0.06	0.07 ± 0.02	0.09 ± 0.01	0.12 ± 0.02	0.04 ± 0.00				
2	PC-3	1,3,4,6,9,15,17		0.20 ± 0.03	0.33 ± 0.07	0.08 ± 0.02	0.13 ± 0.02	0.11 ± 0.03		0.03 ± 0.00		0.33 ± 0.05	
3	Du-145	1,3,4,6,9,15,16		0.36 ± 0.04	0.28 ± 0.05	0.14 ± 0.01	0.17 ± 0.02	0.22 ± 0.02		0.04 ± 0.01	0.09 ± 0.02		
4	TRAMP C-2	1,3,4,6,9,15		0.27 ± 0.09	0.37 ± 0.08	0.04 ± 0.02	0.13 ± 0.02	0.06 ± 0.00		0.05 ± 0.02			
5	TRAMP C-1	1,3,4,6,9,15		0.27 ± 0.07	0.51 ± 0.02	0.06 ± 0.01	0.09 ± 0.01	0.04 ± 0.00		0.05 ± 0.01			
6	Lovo	1,3,4,6,9,15	Colon cancer	0.14 ± 0.07	0.39 ± 0.10	0.15 ± 0.05	0.09 ± 0.02	0.21 ± 0.08		0.06 ± 0.04			
7	HT29	1,3,4,6,9,10,15,16,17		0.38 ± 0.05	0.62 ± 0.12	0.20 ± 0.04	0.21 ± 0.05	0.15 ± 0.03	0.03 ± 0.00	0.05 ± 0.01	0.06 ± 0.01	0.36 ± 0.10	
8	CaCO2	PDI: 1,3,4,6,9,10,16		0.20 ± 0.03	0.27 ± 0.01	0.09 ± 0.01	0.19 ± 0.01	0.17 ± 0.04	0.03 ± 0.00		0.09 ± 0.00		
9	5637	PDI:1,3,4,6,9,10	Urinary/bladder cancer	0.26 ± 0.08	0.35 ± 0.06	0.15 ± 0.02	0.11 ± 0.02	0.06 ± 0.01	0.02 ± 0.00				
10	**MDA MB 231**	PDI:1,3,4,6,9,15	Breast cancer	**0.14 ± 0.03**	**0.17 ± 0.09**	0.10 ± 0.04	0.16 ± 0.07	0.13 ± 0.03		0.05 ± 0.02			
11	67NR	PDI:1,3,6,15,16		0.13 ± 0.01	0.15 ± 0.03		0.14 ± 0.00			0.04 ± 0.00	0.12 ± 0.05		
12	**MCF-7**	PDI:1,3,4,6,9,10,15,17,18		**0.24 ± 0.09**	**0.13 ± 0.04**	0.08 ± 0.01	0.15 ± 0.02	0.21 ± 0.05	0.04 ± 0.01	0.03 ± 0.00		**0.25 ± 0.12**	0.13 ± 0.03
13	4T1	PDI:1,3,4,6,15		0.10 ± 0.03	0.33 ± 0.06	0.03 ± 0.00	0.18 ± 0.03			0.05 ± 0.01			
14	T47D	PDI:1,3,4,6,9,15,16,17		0.53 ± 0.09	0.39 ± 0.13	0.04 ± 0.02	0.10 ± 0.00	0.23 ± 0.02		0.05 ± 0.02	0.08 ± 0.00	0.63 ± 0.19	
15	LLC	PDI:1,3,4,6,15	Lung cancer	0.09 ± 0.03	0.18 ± 0.12	0.02 ± 0.00	0.10 ± 0.02			0.04 ± 0.00			
16	NCI H1703	PDI:1,3,4,6,9,15,16		0.19 ± 0.17	0.23 ± 0.10	0.04 ± 0.02	0.11 ± 0.02	0.15 ± 0.04		0.04 ± 0.00			0.12 ± 0.00
17	A549	PDI:1,3,4,6,9,15,17		0.15 ± 0.01	0.14 ± 0.01	0.05 ± 0.01	0.15 ± 0.02	0.06 ± 0.01		0.03 ± 0.00		0.06 ± 0.00	
18	A427	PDI:1,3,4,6,9,15,16		0.14 ± 0.04	0.20 ± 0.03	0.11 ± 0.05	0.21 ± 0.01	0.07 ± 0.04		0.02 ± 0.00	0.07 ± 0.00		
19	NCI-H358	PDI:1,3,4,6,9,15,16,17,18		0.28 ± 0.12	0.26 ± 0.02	0.07 ± 0.00	0.15 ± 0.02	0.18 ± 0.06		0.04 ± 0.01	0.07 ± 0.00	0.14 ± 0.07	0.12 ± 0.00
20	NCI H1299	PDI: 1,3,6,9,15,16		0.02 ± 0.00	0.11 ± 0.01		0.08 ± 0.02	0.09 ± 0.01		0.03 ± 0.00	0.07 ± 0.00		
21	A2780	PDI:1,3,4,6,9,15,16	Ovarian cancer	0.10 ± 0.02	0.32 ± 0.11	0.12 ± 0.03	0.15 ± 0.01	0.10 ± 0.03		0.06 ± 0.00	0.13 ± 0.00		
22	HT1080	PDI: 1,3,4,6,9,15,16	Fibrosarcoma	0.19 ± 0.03	0.27 ± 0.04	0.17 ± 0.03	0.17 ± 0.03	0.10 ± 0.03		0.04 ± 0.01	0.08 ± 0.00		

The relative abundance of PDIA isoforms (emPAI%) was calculated by dividing the emPAI value for single protein by the sum of all emPAI values for all proteins detected in the sample and presented as a percentage to the overall composition with SEM. The two cell lines selected for further studies (MDA-MB-231 and MCF-7) and their PDIA1, 3 and 17 emPAI% values were highlighted in bold.

**Table 2:** PDI repertoire in the medium of MCF-7 and MDA-MB-231 cell lines

Type of cell	PDIA1	PDIA3	PDIA6	PDIA10
MDA-MB-231	4.740 ± 0.00	8.20 ± 0.57	6.96 ± 0.00	8.16 ± 0.00
MCF-7	2.37 ± 0.00	7.50 ± 0.63	30.70 ± 0.00	2.81 ± 0.00

The average MS signal [×  10^6^] response of the three most intense tryptic peptides with SEM

Interestingly, PDIA17 was expressed in some cancer cell lines (e.g., MCF-7, PC-3, HT-29, T47D, A549, NCIH358) with comparable expression levels to that of PDIA1 and PDIA3. However, PDIA17 was not detected in other investigated cell lines (LNCaP, Du-145, TRAMPC2, TRAMPC1, HT-1080, 4T1, Lovo, 5637, LLC, NCIH1703, A427, NCIH1299, CaCO2, MDA-MB-231).

**Fig. 1:**
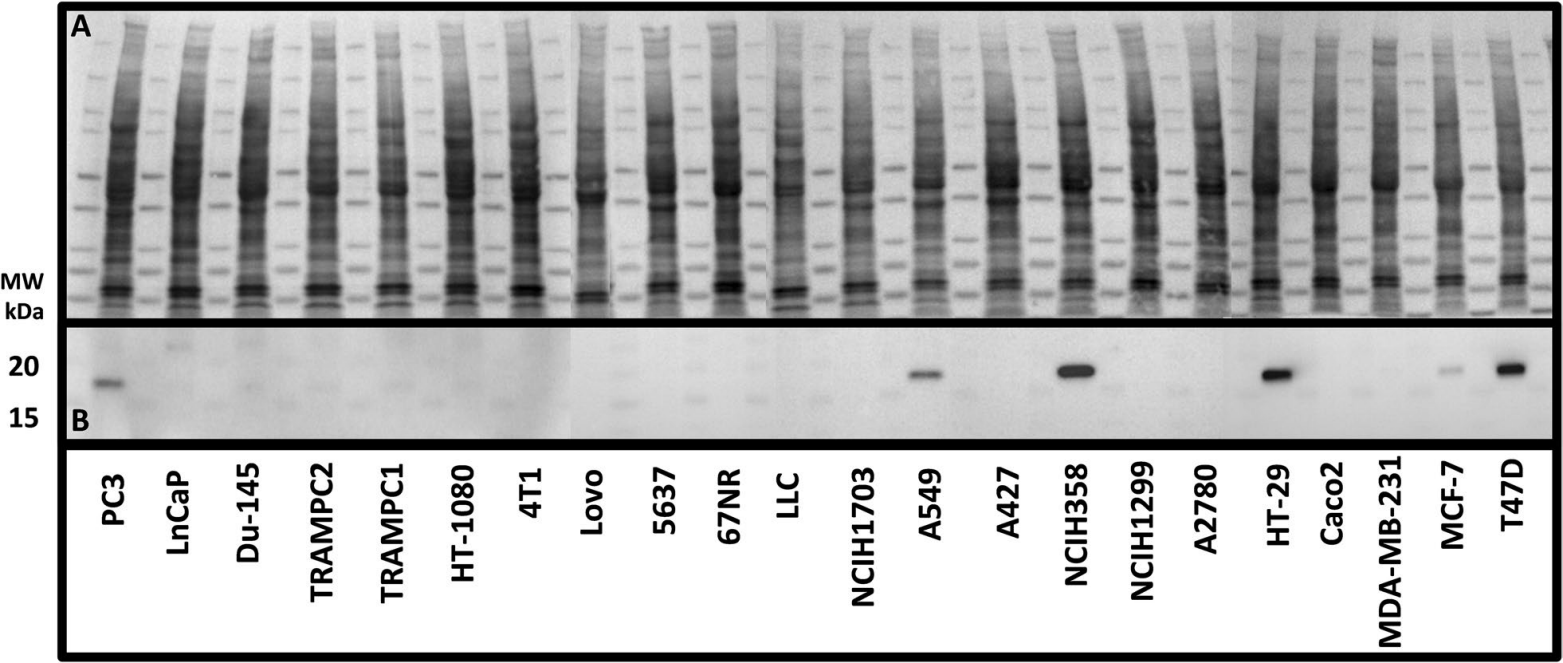
Western blot analysis of PDIA17 in cell lysates of investigated cell lines. **A** Total Protein Staining, **B** PDIA17 expression lane

**Fig. 2:**
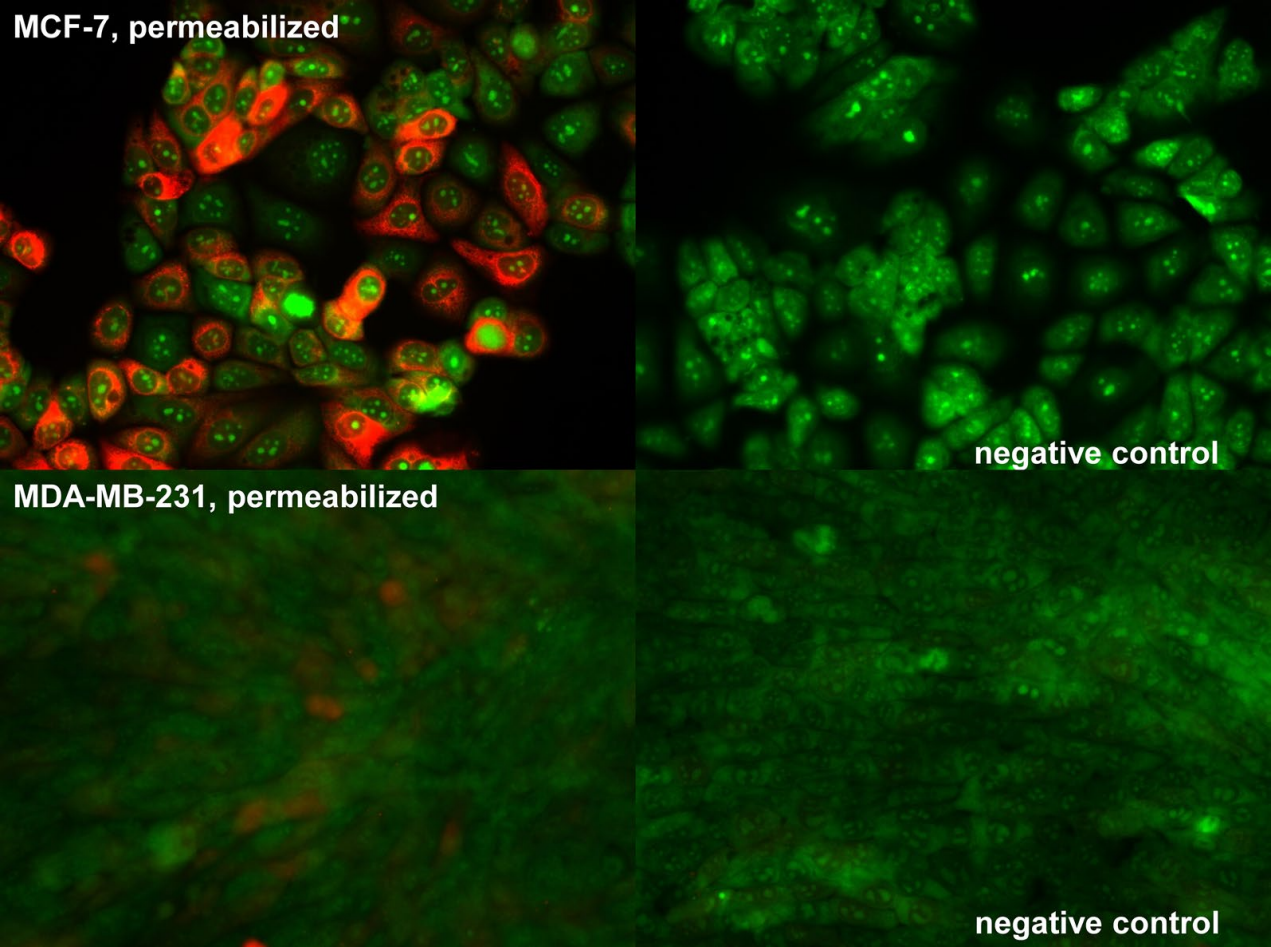
Distribution of PDIA17 inside MCF-7 and MDA-MB-231 cell lines

The presence of PDIA17 was confirmed by Western blot analysis in MCF-7, PC-3, HT-29, T47D, NCIH358 and A549 cell lines (Fig. 1). The immunocytochemistry of the PDIA17 in the two selected cancer cell lines also showed that the expression of PDIA17 was high in MCF-7 and very low in the MDA-MB-231 cell line (Fig. 2).

Based on these results, the MCF-7 cell line was considered to produce a relatively high expression of PDIA17, whereas the MDA-MB-231 cell line was regarded as displaying a low expression of PDIA17. PDIA1 expression was slightly higher in MCF-7 when compared to the MDA-MB-231 cell line (0.24% ± 0.09% and 0.14% ± 0.03%, respectively). PDIA3 expression was similar in both cell lines (0.13% ± 0.04% and 0.17% ± 0.09%, respectively).

### Effects of simultaneous inhibition of PDIA1 and PDIA3 by C-3380, C-3389 and C-3399 on proliferation and adhesion to collagen of MCF-7 and MDA-MB-231 breast cancer cells

**Fig. 3:**
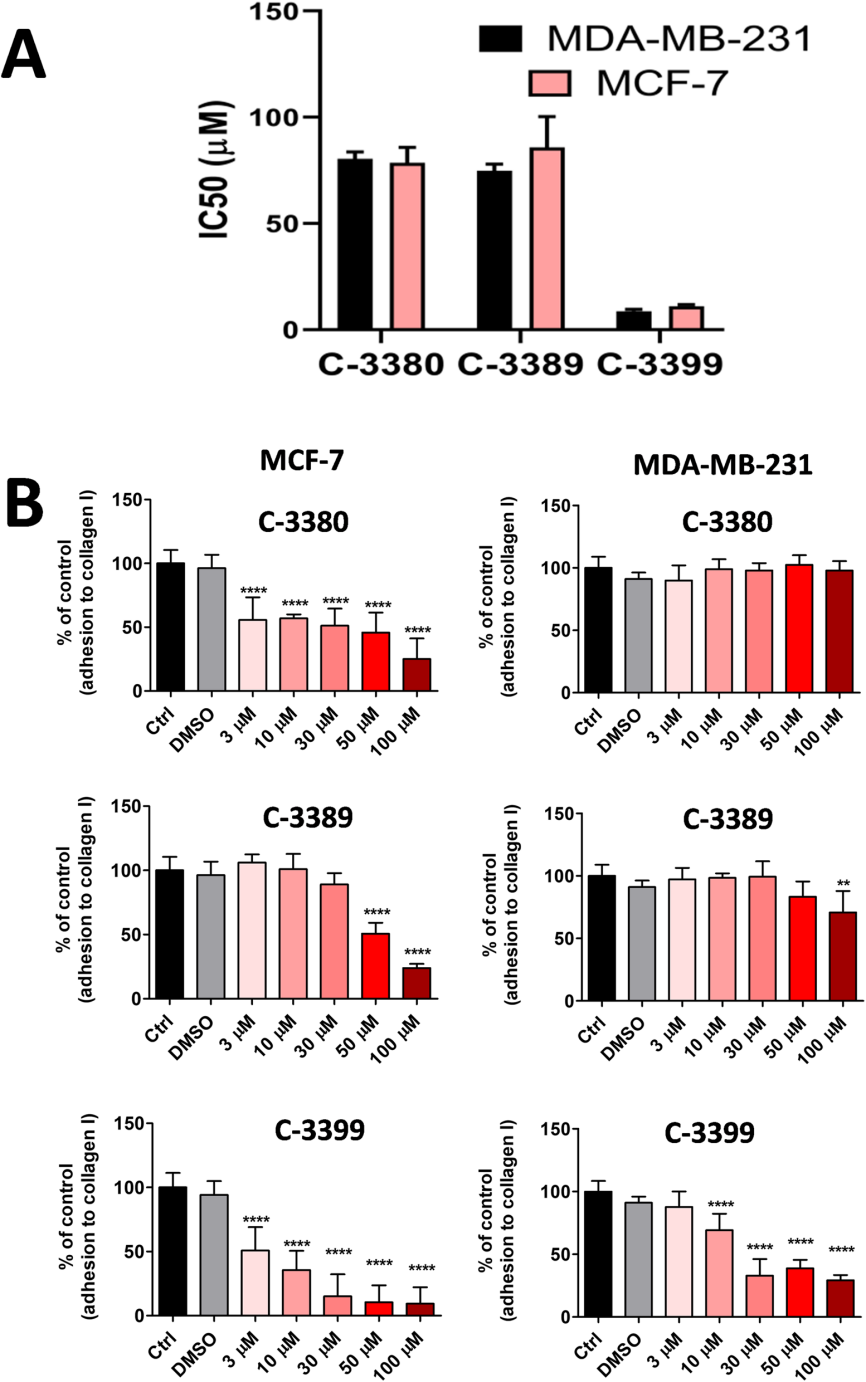
Effects of PDIA1 and PDIA3 inhibitors (C-3380, C-3389, C-3399) on breast cancer cell proliferation and adhesion in PDIA17 low and high expressing cell line (MDA-MB-231 vs MCF-7, respectively). **A** Half-maximal inhibitory concentration (IC_50_) for PDIA1 and PDIA3 inhibitors in viability test in breast cancer cell lines; **B** Effects of PDIA1 and PDIA3 inhibition on adhesion of breast cancer cell lines to collagen, type I

As shown in Fig. 3A, the anti-proliferative effects for C-3380, C-3389 and C-3399 were similar in the MCF-7 and MDA-MB-231 cell lines. IC_50_ values for C-3380 reached 80.45  ± 3.18 µM in MDA-MB-231 and 78.61 ± 7.20 µM in the MCF-7 cell line. For C-3389, the IC_50_ was 74.77 ± 3.17 µM in MDA-MB-231 and 85.86  ± 14.5 µM in MCF-7, whereas the IC_50_ of C-3399 was lower: 8.69  ± 0.89 µM in MDA-MB-231 and 11.06  ± 0.78 µM in MCF-7.

In contrast to the similar anti-proliferative effects of C-3380, C-3389 and C-3399 inhibitors, the inhibition of adhesion of MCF-7 cells to collagen by these compounds was more pronounced as compared to the MDA-MB-231 cells. The adhesion of MCF-7 cells to the collagen was significantly inhibited (ca*.* 50% of control) by C-3380 and C-3399 at a concentration of 3 µM and by C-3389 at a concentration of 50 µM. In turn, the adhesion of MDA-MB-231 to collagen was reduced (ca*.* 75% of control) by C-3399 at a concentration of 10 µM and weakly by C-3389 at a higher concentration (100 µM) (Fig. 3B).

### Effects of simultaneous inhibition of PDIA1 and PDIA17 by C-3353 on proliferation and adhesion of MCF-7 and MDA-MB-231 breast cancer cells

**Fig. 4:**
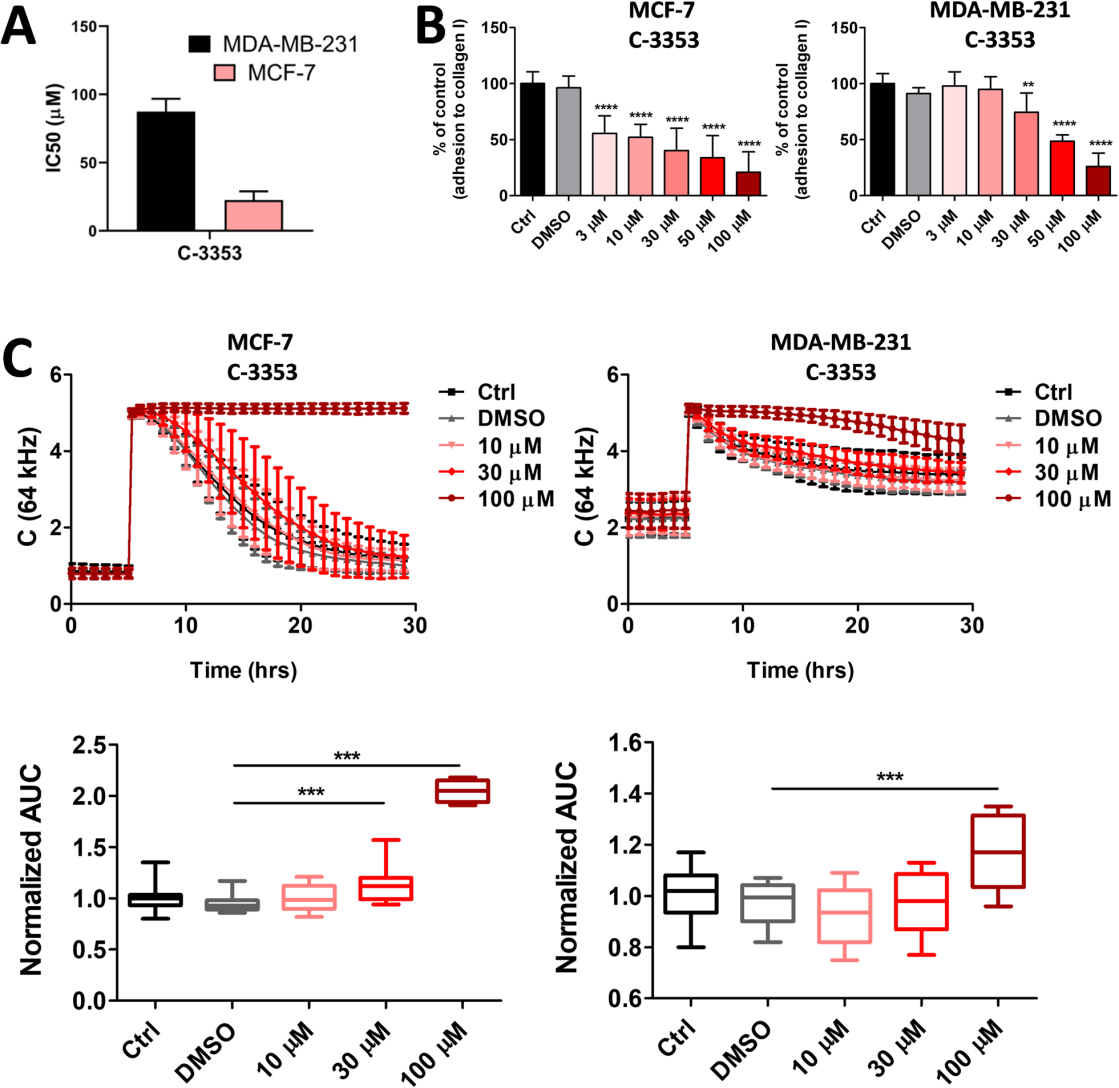
Effects of parallel PDIA17 and PDIA1 inhibition (C-3353) on breast cancer cell proliferation, migration and adhesion in PDIA17 low and high expressing cell line (MDA-MB-231 vs MCF-7, respectively). **A** Half-maximal inhibitory concentration (IC_50_) for PDIA17 and PDIA1 inhibitor in viability test in breast cancer cell lines; **B** Effects of parallel PDIA17 and PDIA1 inhibition on adhesion of breast cancer cell lines to collagen, type I; **C** Effects of parallel PDIA17 and PDIA1 inhibition on wound-healing and migration of breast cancer cells

The anti-proliferative effects of C-3353 in the MTT assay were more pronounced in the MCF-7 cell line with high expression of PDIA17 when compared to the MDA-MB-231 cell line with low expression of PDIA17 (22.51  ±  6.42 µM and 87.41  ±  9.42 µM, respectively) (Fig. 4A). Similarly, in the electric cell-substrate impedance-sensing (ECIS)-based wounding assay, the migration rate in the MCF-7 cell line was substantially reduced by C-3353 at a concentration of 30 µM or higher, whereas the recovery was rapid in the MDA-MB-231 cell line, and the inhibition by C-3353 was effective only at a concentration of 100 µM (Fig. 4B).

Similarly, C-3353 inhibited the adhesion of MCF-7 cells to the collagen at a lower concentration (3 µM) as compared with MDA-MB-231 cell adhesion, which was inhibited by C-3353 at a concentration of 50 µM (Fig. 4C).

### Cytotoxicity of the compounds against regular cell lines

To test cytotoxicity of compounds used in this study against regular cell lines, BALB/3T3 and MCF10A cells were used, mouse fibroblast and human breast epithelial cell line, respectively. In BALB/3T3 cells the IC_50_ values for C-3380, C-3389, C-3399 and C-3353 were 90.89  ±  17.18 µM, 34.34  ±  13.09 µM, 38.16  ±  10.33 µM and higher than 360 µM, respectively. In MCF10A cells, IC50 values were: 59.29  ±  8.3 µM, 20.42  ±  5.35 µM, 17.85  ±  1.15 µM and 75.60  ±  4.05 µM, respectively.

## Discussion

In the present work, we characterized PDI isoform contents in various cancer cell lines and provided evidence that PDIA1 and PDIA3 represent major isoforms of multiple cancer cells, and the non-selective inhibition of PDIA1 and PDIA3 displayed significant anti-proliferative effects irrespective of whether or not PDIA17 was present. On the other hand, PDIA17 was highly expressed in the hormone-sensitive MCF-7 breast cancer cell line when compared to the triple-negative MDA-MB-231 cell line, and PDIA17 inhibition displayed additional anti-proliferative effects in the MCF-7 but not in the MDA-MB-231 breast cancer cell line. Interestingly, the anti-adhesive effects in the hormone-sensitive MCF-7 cells featured by higher levels of PDIs were clearly more pronounced when compared to triple-negative MDA-MB-231 cells, suggesting that PDIA1-, PDIA3- and PDIA17-based therapy may represent a novel strategy for personalized anti-metastatic therapy in cancers with high PDI expression.

In previous studies, the overexpression of the PDIs in comparison to normal tissue was demonstrated (e.g., using RNA-Seq methodology), and there is a general agreement on the important role of PDIs in cancer survival [29]. The data from gene expression datasets showed that the expression levels of individual PDI members vary between the types of cancer. Higher expression of PDIA1 was, for example, noted in prostate, ovarian and oesophageal cancer [29]. However, to the best of our knowledge, our study is the first to comprehensively characterize the content of various PDI isoforms in multiple cancer cell lines using a proteomic approach to determine which of these isoforms are the most abundant and might represent an optimal target for anti-cancer therapy.

The major contribution of PDIA1 and PDIA3 to the total PDI profile of various cancer cells, their secretion and important role outside the cell agrees with growing evidence for the involvement of PDIA1 and PDIA3 in cancer progression [16, 30–35].

Importantly, independent of this general rule, the profile of individual PDIs did vary to some extent between cancer types (e.g*.,* prostate, breast cancer) and cell lines within the same type of cancer. For example, among lung cancers, the NCIH1299 cell line had very low expression of PDIA1, whereas in the NCIH358 cell line, this expression was 14 folds higher. In particular, in breast cancer cell lines, PDIA17 expression was high in the hormone-sensitive MCF-7 cell line but not detectable in the triple-negative MDA-MB-231 cell line. This was an important finding of our work in the context of the suggested role of PDIA17 in genomic integrity, proliferation, apoptosis, angiogenesis, adhesion, migration, stemness and inflammation [18, 36], indicating that PDIA17 is a promising target for anti-cancer treatment. In fact, the high expression of PDIA17 in the primary tumour of breast cancer patients was found to be a possible prognostic indicator of poor patient outcome [37]. Increased plasma concentration of PDIA17 was positively correlated with cancer progression [38]. In our hands, this protein was expressed only in a few studied cell lines, including PC-3, MCF-7, HT-29 and T47D, thus forming an anti-cancer target for selected cancers. Unexpectedly, higher PDIA17 expression was noted in the less-invasive breast cancer cell line [3, 39], suggesting the importance of hormonal sensitivity or involvement of other regulatory mechanisms [10, 40–42]. Interestingly, in breast cancers, the rationale for anti-PDIA17 therapy was offered for oestrogen receptor (ER)-positive breast cancers (e.g., MCF-7) but not ER-negative breast cancers (e.g., MDA-MB-231) [43–45].

In the context of the emerging literature on the role of PDIA17 in tumorigenesis, based on our proteomic analysis confirmed by Western blotting, we were able to clearly define MCF-7 as a breast cancer cell line with high PDIA17 expression, whereas MDA-MB-231 had very low PDIA17 expression, and we used these two cell lines for pharmacological studies. Importantly, the intercellular expression of PDIA1 and PDIA3 in these two cell lines displayed only modest differences in their relative intracellular abundance; therefore, these two lines were well suited to delineate the relative functional importance of PDIA1 and PDIA3 as compared with PDIA17. As pharmacological tools to inhibit PDIA1, PDIA3 or PDIA17, we used recently developed aromatic N-sulphonamides of aziridine-2-carboxylic acid derivatives [19, 20]. These compounds represent a novel group of potent and relatively selective PDIA1 inhibitors with additional activity towards PDIA3. Many of them show low or moderate cytotoxic activity in vitro towards a panel of cancer cell lines that was for numerous compounds more pronounced as compared with their cytotoxicity against normal cell lines [19, 20]. However, their anti-cancer effects have not been previously investigated in the context of heterogeneous contents of PDIs in various cancer cell lines.

Here, we demonstrated in the anti-proliferative assays that MCF-7 and MDA-MB-231 exhibited similar responses to the PDI inhibitors used, suggesting that targeting the most abundant PDI isoforms regardless of PDIA17 levels may be an effective strategy to inhibit the proliferation of cancer cells.

In turn, the anti-adhesive properties of the tested PDIA1 and PDIA3 inhibitors were more pronounced in the MCF-7 cell line, a result that might be attributed to higher expression of PDIA1 on the surface membrane of the cells in comparison to the MDA-MB-231 cell line because extracellular PDIA1 seemed more abundant in the cell line. The role of PDIA1 in cancer cell adhesion was in accordance with our recent work demonstrating that extracellular PDIA1 plays an important role in regulating the adhesion of cancer cells to matrix proteins, endothelium and their transendothelial migration [11]. Similar extracellular mechanisms of action could be attributed to PDIA3. Indeed, PDIA3 was also present extracellularly in both MCF-7 and MDA-MB-231 cell lines. The compound C-3399, which inhibited PDIA1 and PDIA3 rather unselectively, with some preferences to the inhibition of PDIA3 was the most potent inhibitor of adhesion of MDA-MB-231 cells to collagen, underscoring the therapeutic value of non-selective inhibition of PDIA3 and PDIA1 in the regulation of cancer cell adhesion to the cellular matrix. The relative importance of PDIA3 vs PDIA1 in this effects remains to be delineated in further studies. Finally, we demonstrated that C-3353 inhibition of PDIA17 was quite potent in inhibiting the adhesion of MCF-7 to collagen. Altogether, our results show that targeting PDIA1 and PDIA3 as well as PDIA17 might afford effective anti-cancer effects, particularly in highly malignant cancer cell types by targeting cancer cell adhesion. Such a mechanism of action involving extracellular disulphide exchange and most likely integrin activation might afford important anti-metastatic effects independent of cytostatic or anti-proliferative potential of PDI inhibition [11]. Furthermore, it seems that effects of the tested PDI inhibitors cannot be ascribed to apoptosis. The studies of Stojak et al. [11] showed that the PDI inhibition was not accompanied by increased caspase 3/7 activity in MDA-MD-231 cand MCF-7 cell lines. This conclusion was confirmed by using the two strategies of PDI inhibition–PDIA1 and PDIA3 silencing and bepristat treatment. Additionally, the authors did not observe changes in mitochondrial respiration and only moderate changes in cell cycle. An increase in caspase 3/7 activity was noted in the studies of the effects of other azaridine-based PDI inhibitors in colon cancer, but only with the highest concentration used (data not published). In contrast, in reports using cell permeable PDI-inhibitors e.g. BAP2 and 35G8 the activation of apoptosis and autophagy were reported [46] the effects most likely linked to ER stress and the unfolded protein response (UPR) [3]. Accordingly, we claim that PDI inhibitors that do not penetrate across the cell membrane would target extracellular PDIs and their action is not necessarily associated with caspase inhibition but affords the inhibition of cancer cell adhesion to the cellular matrix or to endothelial cells. Indeed, membrane-impermeant thiol blocker pCMBS, inhibited cancer cell adhesion to collagen type I and fibronectin [11] supporting the notion that extracellular PDIs is a target for anti-adhesive effects of PDIA1 inhibitors.

Noteworthy, the majority of available inhibitors of PDIs are non-selective sulfhydryl-reactive compounds that mostly target intracellular PDIs. For example, the irreversible PDIA1 inhibitor—PACMA31—efficiently suppressed ovarian tumour growth, suggesting non-selective intracellular PDI inhibition as a major target for anti-cancer PACMA 31 action [10, 47]. Similarly, a small molecule intracellular inhibitor of PDI activated the apoptosis signalling pathway and reduced the malignancy of colorectal cancer [48]. CCF642 caused acute ER stress in multiple myeloma cells accompanied by apoptosis in addition to a robust ER stress response [49]. E64FC26 induced the induction of oxidative stress in multiple myeloma [50]. All these mechanisms involved intracellular targets, and the compounds used were cell-permeable and unselective PDI inhibitors. The development of effective and selective cell-impermeable PDI inhibitors may pave the way for further studies targeting cancer cell adhesion and metastasis by targeting extracellular PDIs.

## Conclusions

In conclusion, PDIA1 and PDIA3 represent the most abundant isoforms in multiple cancer cell lines and are also bound to membranes and released extracellularly providing the rationale of the development of anti-PDIA1 and anti-PDIA3 therapy targeted to extracellular PDIs. PDIA17 expression appears to be cell specific and related to cancer cell malignancy, and its inhibition could present additional benefits in cancer cells expressing PDIA17. In particular, it seems that PDIA17 targeting might be of importance in some ER-positive cancers. Targeting extracellular PDIA1-, PDIA- and PDI17-dependent regulation of cancer cell adhesion may represent a novel, effective, personalized anti-adhesive and anti-metastatic therapy in cancers with high PDI expression. This notion agrees with an increasing interest in the molecular mechanisms of action of extracellular PDIs [51]. Further studies are required to explore the extracellular PDIs as anti-cancer targets.

## Supplementary Information

**Additional file 1: Methods.** Contains all additional information on methods used in the study.

**Additional file 2: Table S1.** The origin and conditions of maintenance of cell cultures.

**Additional file 3: Table S2.** The half maximal inhibitory concentration for selected PDIA1, PDIA3 and PDIA17 inhibitors.

## Data Availability

The datasets generated during and/or analysed during the current study are available from the corresponding author on reasonable request.

## Abbreviations

ABC: Ammonium bicarbonate
ACN: Acetonitrile
AUC: Area under the curve
BSA: Bovine serum albumin
DMSO: Dimethyl sulfoxide
DPBS: Dulbecco's phosphate-buffered saline
DTT: DL-dithiotreitol
ECIS: Electric cell-substrate impedance-sensing assays
emPAI: Exponentially modified protein abundance index
ER: Estrogen receptor
FA: Formic acid
HCD: High collision dissociation
IAA: Iodoacetamide
MOM blocking reagent: Mouse on mouse blocking reagent
PDI: Protein disulphide isomerase
PDIA1: Protein disulphide isomerase A1 (beta-subunit of prolyl 4-hydroxylase, P4HB)
PDIA3: Protein disulphide isomerase A3 (endoplasmic reticulum resident protein 57, ERp57)
PDIA4: Protein disulphide isomerase A4 (endoplasmic reticulum resident protein 72, ERp72)
PDIA6: Protein disulphide isomerase A6 (endoplasmic reticulum protein 5, ERp5)
PDIA9: Protein disulphide isomerase A9 (endoplasmic reticulum resident protein 29, ERp29)
PDIA10: Protein disulphide isomerase A10 (endoplasmic reticulum resident protein 44, ERp44)
PDIA15: Protein disulphide isomerase A15 (thioredoxin domain-containing protein 5, TXNDC5)
PDIA16: Protein disulphide isomerase A16 (thioredoxin domain-containing protein 12, TXNDC12)
PDIA 17: Protein disulphide isomerase A17 (anterior gradient protein 2, AGR2)
PDIA18: Protein disulphide isomerase A4 (anterior gradient protein 3, ARG3)
PIC: Protease and phosphatase inhibitor cocktail
PR: Progesterone receptor
PVDF membranes: Polyvinylidene membranes
RT: Room temperature
SRB: Sulforhodamine B
TFA: Trifluoroacetic acid
UPR: Unfolded protein response
