# Supplementary material for: Comparison of anti-cancer effects of novel protein disulphide isomerase (PDI) inhibitors in breast cancer cells characterized by high and low PDIA17 expression
Source: Cancer Cell Int. 2022 Jun 20;22:218. doi: 10.1186/s12935-022-02631-w (PMC9208212; doi:10.1186/s12935-022-02631-w)
Supplement: Supplementary file 1 — Additional file 1: Methods. Contains all additional information on methods used in the study. [file 12935_2022_2631_MOESM1_ESM.docx]

**Comparison of anti-cancer effects of novel protein disulphide isomerase (PDI) inhibitors in breast cancer cells characterized by high and low PDIA17 expression**

Kurpińska Anna^1^, Suraj-Prażmowska Joanna^1^, Stojak Marta^1^, Jarosz Joanna^2^, Mateuszuk Łukasz^1^, Niedzielska-Andres Ewa^3^, Smolik Magdalena^3^, Wietrzyk Joanna^2^, Kalvins Ivars^4*^, Walczak Maria^1,3*^, Chłopicki Stefan^1,5*^

^1^ Jagiellonian University, Jagiellonian Centre for Experimental Therapeutics (JCET), Bobrzynskiego 14, 30-348, Krakow, Poland

^2^ Hirszfeld Institute of Immunology and Experimental Therapy, Department of Experimental Oncology, Polish Academy of Sciences, Rudolfa Weigla 12, 53-114, Wroclaw, Poland.

^3^ Jagiellonian University Medical College, Faculty of Pharmacy, Chair and Department of Toxicology, Medyczna 9, 30-688, Krakow, Poland

^4^ Latvian Institute of Organic Synthesis, Laboratory of Carbofunctional Compounds, LV-1006, Riga, Latvia

^5^ Jagiellonian University Medical College, Faculty of Medicine, Chair of Pharmacology, Grzegorzecka 16, 31-531, Krakow, Poland

*Corresponding authors: Stefan Chlopicki, stefan.chlopicki@jcet.eu, Maria Walczak, maria.walczak@jcet.eu, Ivars Kalvins, ivars.kalvins@lza.lv

**Additional file 1: Methods**

**Preparation of medium for proteomic analyses**

The medium was lyophilized, resuspended in 1 ml of MilliQ water, and 10 mg protein was processed using a ProteoMiner Protein Enrichment Kit (Bio-Rad, Hercules, USA) according to the procedure of Boschetti and Righetti (2013). Briefly, 20 µl of settled beads were used, which referred to 100 µl of a 20% aqueous ethanol bead slurry. The number of beads used was calculated based on the recommended ratio: 0.5 mg protein per 1 µl of beads. After removal of the bead slurry, the beads were washed with water, equilibrated three times with 25 mM phosphate buffer at pH 7.2 containing 50 mM NaCl (VWR, Radnor, USA), and the medium was incubated with beads for 3 h on a rotational shaker. The liquid was depleted, and the beads were washed in triplicate with 25 mM phosphate buffer used previously for equilibration. The proteins were detached from beads using double incubation with 4% SDS (Poch, Gliwice, Poland) with 25 mM DL- dithiothreitol (DTT; Bio-Shop, Burlington, Canada) (99 °C, 5 min) (Boschetti and Righetti, 2013). For further proteomic analysis, the obtained eluent was incubated overnight with four volumes of ice-cold acetone. After centrifugation (15000 x g, 30 min., 0°C), the pellet was dissolved in 50 mM ammonium bicarbonate (ABC) (Sigma-Aldrich, Saint Louis, USA). The protein concentration in the medium processed using the ProteoMiner Protein Enrichment Kit was assessed each time using a Bradford assay (Bio-Rad, Hercules, USA).

**Confirmation of PDIA17 presence or absence in cell lysates using Western blots**

Lysates of all cell lines used in the study were checked for PDIA17 presence using Western blotting. Samples were diluted with PBS with a commercially available protein inhibitor cocktail (Sigma-Aldrich, Saint Louis, USA) to the same total protein concentration and mixed with loading buffer (Laemmli buffer 2x concentrate, Sigma-Aldrich, Saint Louis, USA) and then heated for 10 min at 95 °C. Afterwards, the samples were run on gradient 4–15% SDS polyacrylamide gels (Bio-Rad, Hercules, USA) under reducing conditions and electrophoretically transferred onto (polyvinylidene) PVDF membranes (Thermo Fisher Scientific, Waltham, USA). Blots were blocked with 5% non-fat dry milk and incubated with rabbit recombinant monoclonal anti-anterior gradient 2 antibody (ab209224, Abcam, Cambridge, UK) and then with secondary goat anti-rabbit antibody (ab7090, Abcam, Cambridge, UK). Obtained blots were visualized by chemiluminescence utilizing a Western Bright Quantum ECL western blotting kit (Advansta, San Jose, USA) according to the manufacturer's instructions with the aid of a G:BOX Chemi Imaging System controlled by Gene Tools software. Finally, the results were standardized with total protein staining as the loading control using a Pierce™ Reversible Protein Stain Kit for PVDF Membranes (Thermo Fisher Scientific, Waltham, USA).

**Immunocytochemistry of PDIA17 in MDA-MB-231 and MCF-7 cell lines**

The cells MCF-7 and MDA-MB-231 were stained using immunocytochemistry. Complete information on the general cell culture conditions is provided in **section** **2.1.** The cells that were selected for further investigation were seeded in 96-well format using black corning plates with a transparent bottom. Next, the cells were washed with PBS, fixed with 4% formalin solution (10 min) (Sigma-Aldrich, Saint Louis, USA), again washed with PBS, roughly permeabilized using 0.1% Triton-X (5 min) (Sigma-Aldrich, Saint Louis, USA) and then preincubated for 30 min with blocking buffer with 5% normal goat serum (Jackson Immuno, Ely, UK) and 2% dry milk. Immunofluorescent staining was performed for 1 h (overnight for PDIA17 of HT-29 due to the different dilutions of primary antibodies) using rabbit anti-anterior gradient 2 Ig (ab209224, Abcam, Cambridge, UK), followed by 30 minutes’ incubation with Cy3-conjugated goat-anti-rabbit secondary antibodies (111-165-144, Jackson Immuno, Ely, UK). Cells stained with mouse antibodies were previously incubated with mouse-on-mouse (MOM) blocking reagent (Vector, Burlingame, USA) to reduce unspecific binding. Hoechst 33258 (Sigma-Aldrich, Saint Louis, USA) was used for nuclei counterstaining. Cell images were taken using an AxioObserver.D1 inverted fluorescent microscope and monochromatic AxioCam HRm digital camera (Carl Zeiss, Oberkochen, Germany), stored as integrated datasheets and analyzed automatically using Columbus software (Perkin Elmer, Waltham, USA).

**Synthesis of aromatic N-sulphonamides of aziridine-2-carboxylic acid derivatives, PDIA1, PDIA3 and PDIA17 inhibitors**

Details on the structure and synthesis of novel PDIA1 and PDIA3 inhibitors (C-3380, C-3389, C-3399) and C-3353 that also displayed PDIA17 inhibition properties were described previously (Kalvins et al. 2021; Chlopicki et al., 2021). Briefly, for PDI inhibitor synthesis, aromatic sulphonic acid chloride (1 mmol) (Sigma-Aldrich, Saint Louis, USA) was added with stirring to the solution of the appropriated aziridine (1.1 mmol) and K_2_CO_3_ (2 mmol) (Sigma-Aldrich, Saint Louis, USA) in the mixture of 1 ml CHCl_3_ (Sigma-Aldrich, Saint Louis, USA)+ 1 ml water. The mixture was stirred for 24 h at RT. The product was extracted with CHCl_3_ (Sigma-Aldrich, Saint Louis, USA) and the solution dried over MgSO_4_ (Sigma-Aldrich, Saint Louis, USA). The solvent was evaporated. The product was purified by liquid chromatography (silica gel, petroleum ether/ethyl acetate 4:1=>1:2) to give corresponding aziridine aromatic N-sulphonamide as described previously (Kalvins et al. 2021; Chlopicki et al., 2021).

The PDI inhibitors’ activity towards PDIA1, PDIA3 and PDIA17 was assessed as an increase in disulphide bond reduction in human insulin in the presence of DTT causing aggregation of its β-chain, analyzed by turbidimetry as presented earlier by Kalvins et al. 2021 and Chlopicki et al., 2021. Briefly, the assay mixture for 96-well plates was prepared by dissolving in 0.1 mM phosphate buffer (pH 7.6 for activity testing of PDIA1, PDIA3 and pH 8.0 for PDIA17), 6 µg/ml (96 nM) of PDIA1 (recombinant protein MBS9422429, human, full length, 18-508aa His-tag, *E.coli,* MyBioSource, San Diego, CA, USA), PDIA3 (recombinant protein MBS203583, 25-505aa, human, His-tag, *E.coli*, MyBioSource, San Diego, CA, USA) or PDIA17 (recombinant protein ab64013, human, full length, *E.coli*, Abcam, Cambridge), 2 mM EDTA and 0.08 mM DTT (D9779 Sigma-Aldrich, Steinheim, Germany). Stock solutions of C-3353, C-3380, C-3389 and C-3399 were freshly prepared in dimethyl sulfoxide (DMSO) and subsequently diluted to keep the final DMSO concentration in the assay mixture below 1%. Calculated amounts of compounds solutions were added into test wells, and the reaction was started by the addition of insulin (insulin 91077C, human recombinant; Sigma-Aldrich, Steinheim, Germany) and 0.08 mM of DTT. The final concentration of insulin and DTT in the assay mixture was 0.15 and 0.16 mM, respectively. The reaction rate was monitored at 650 nm on a Microplate Reader Infinite M1000 PRO for 60 minutes at 37°C. Turbidity values for the wells containing only the PDI isoform tested (background values) were subtracted from the turbidity values of the wells containing the tested PDI isoform and selected inhibitors. The inhibition of the PDI isoform’s catalytic activity in the presence of inhibitors was calculated by the following formula: enzyme inhibition (%) = [1–(OD[inhibitor + PDI + DTT]–OD[DTT])/(OD[PDI + DTT]–OD[DTT])] × 100%, where OD refers to the optical density.

**Semi-quantitative proteomic analysis of PDI contents using mass spectrometry**

The cell lysates and cell medium for the proteomic analysis of PDIs were prepared according to a procedure outlined by Sitek et al. (2012) with slight modifications. For this purpose, 10 µg protein was dissolved in 258 µl 50mM ABC. A reduction and alkylation were performed with 45 mM DTT in 50 mM ABC (15 min., 50°C) and 100 mM iodoacetamide (IAA) in 50 mM ABC (15 min., RT), respectively. Sequencing grade modified trypsin (Promega, Madison, USA) in a 1:50 w/w ratio was used for the digestion of proteins (16 h, 37°C). The digestion was quenched by adding 10 µl formic acid (FA; Merck, Darmstadt, Germany). The samples were centrifuged (16000 x g, 30 min.), and the supernatant was lyophilized.

The proteomic analysis of cell lysates was conducted using a Dionex UltiMate™ 3000 RSLC System (Thermo Scientific, San Jose, USA) coupled to an LTQ XL hybrid ion trap-Orbitrap Discovery mass spectrometer (Thermo Scientific, San Jose, USA) as described by Kurpinska et al. (2019). Briefly, the peptides obtained (10 µL of sample) after the procedure of the sample preparation were trapped at 10 µl/min (loading buffer consisted of 2% acetonitrile (ACN; Witko, Lodz, Poland in H_2_O) and then separated on the analytical column at 250 nl/min (0.1% FA in H_2_O and 0.1% FA in ACN). A gradient-based method was applied. The mass spectrometer was operated in data-dependent mode. A full scan was performed from m/z 300–2000, with resolution R=30000 at 400 m/z. The ten most intense ions were used for fragmentation in a linear ion trap with collision-induced dissociation using helium (He) as a collision gas. The identification of proteins was performed based on the obtained mass spectra, using the MASCOT search engine (MatrixScience, London, UK, Mascot Server 2.5) and the Swiss-Prot database. The following parameters were set: enzyme: trypsin, taxonomy: *Mus musculus/Homo sapiens*, precursor mass tolerance: 10 ppm, fragment mass tolerance 0.1 Da, 1 missed cleavage, fixed modifications: carbamidomethylation of cysteine, dynamic modifications: oxidation of methionine, false discovery rate: 1% (p≤0.01).

For the detection and semi-quantitation of PDI isoforms in cell lysates, the exponentially modified protein abundance index (emPAI) calculation was performed using the in-built tool of the Mascot search engine based on protein coverage by the peptide matches in a database search result. The calculation includes the number of observed vs observable peptides for each protein (Ishihama et al., 2005). The emPAI values were normalized to the total emPAI value—the emPAI value calculated for a single protein was divided by the sum of all emPAI values for all proteins present in the sample and presented as a percentage to the overall composition (Roy et al., 2017). The data for PDIs’ semi-quantitation in cell lysates are presented as mean±SEM.

The repertoire of released PDIs was characterized in the two lines selected for further studies. To define the PDIs secreted by MDA-MB-231 (negative/low PDIA17 expression) and MCF-7 (high PDIA17 expression), cell media were subjected to enhanced proteomic analysis. Peptides were reconstituted in 0.1% trifluoroacetic acid (TFA; Sigma-Aldrich, Saint Louis, USA) with 2% ACN (J.T. Baker, New Jersey, USA) and subjected to LC-MS/MS analysis. Additional mass spectrometry analysis was performed in the Mass Spectrometry Laboratory at the Institute of Biochemistry and Biophysics, Polish Academy of Sciences, Warsaw, Poland. An amount of 10 μl of each sample was analyzed using an LC-MS system composed of a UPLC chromatograph (NanoAcquity, Waters, Milford, USA) directly coupled to a QExactive mass spectrometer (Thermo Fisher Scientific, Waltham USA). Peptides were trapped on a C18 pre-column (ACQ M-Class SYM100 C18 5 μ VM 180 μm x 20 mm, Waters, Milford, USA) using 0.1% FA (Merck, Darmstadt, Germany) in water as a mobile phase and then transferred to a nanoAcquity BEH C18 column (75 µm x 250 mm, 1.7 µm, Waters, Milford, USA) using an ACN gradient (0–35% ACN for 160 min) in the presence of 0.1% FA at a flow rate of 250 nl/min. The column temperature was set to 35°C. Data acquisition was carried out using a data-dependent method with the top 12 precursors selected for MS2 analysis with high collision dissociation (HCD) fragmentation and dynamic exclusion set to 30 s. Full mass spectra were obtained from m/z 300 to 1650 with a resolution of 70 000. MS/MS spectra were acquired at a resolution of 17 500 and a maximum injection time of 60 ms for both MS1 and MS2. The automatic gain control (AGC) target for MS1 was set to 1e6 and the AGC target for MS2 to 2e5. The isolation window was set at 3 m/z and the fixed first mass at 100 m/z. A blank run ensuring an absence of cross-contamination from previous samples preceded each analysis. For protein identification, Proteome Discoverer software (version 1.4, Thermo Scientific, San Jose, USA) was used. The MS/MS-spectra were searched against the Swiss-Prot database with the taxonomic restrictions to the human proteins deposited in Swiss-Prot (20,488 sequences). The following parameters were set: enzyme - trypsin, taxonomy – *Homo sapiens*, precursor mass tolerance - 10 ppm, fragment mass tolerance - 0.1 Da, 1 missed cleavage, fixed modifications - carbamidomethylation of cysteine, dynamic modifications - oxidation of methionine and false discovery rate - 1% (p≤0.01) (Stojak et al. 2020, Malinowska et al., 2012). The data for PDIs present in the medium are presented as the average MS signal response of the three most intense tryptic peptides with SEM (Silva et al., 2006).

**MTT cell viability assay – half-maximal inhibitory concentration (IC_50_) assessment**

The proliferation rate of the cells was measured using an MTT assay. The cells were seeded on 96-well plates (Sarstedt, Warsaw, Poland) at a density of MDA-MB-231, BALB/3T3 and MCF10A 1×10^4^ cells/well, MCF-7 0.75×10^4^ cells/well. Cells were incubated in the appropriate culture medium for 24 h before adding the tested compounds. Cells were then treated with each compound in four concentrations in the range 100–0.1 µg/mL. Cell proliferation was evaluated after 72 h of incubation using an MTT test, and determination of the IC_50_ parameter (i.e., the concentration of the compound that inhibits cell proliferation by 50%) was performed as previously described (Psurski et al., 2017). Absorbance was measured using a Bio Tek Hybrid H4 reader (BioTek Instruments, USA) at 570 nm wavelength.

Compounds at each concentration were tested in triplicate in a single experiment, and each experiment was repeated at least three times independently. Dimethyl sulfoxide (DMSO), used as a stock solution solvent, was tested for anti-proliferative activity and did not affect cell proliferation at 0.1% (v/v)—the highest concentration used in compound solutions.

**Cancer cell adhesion to collagen type I**

The ability of breast cancer cells to adhere to collagen type I was analyzed using a functional adhesion assay according to the procedure reported by Stojak et al. (2020) with minor modifications. Briefly, 96-well plates (Corning, Tewksbury, MA, USA) were coated with collagen type I at a concentration of 10 μg/mL (Sigma-Aldrich, Steinheim, Germany) and incubated at 4°C overnight. After 24 h, the plates were blocked with 1% bovine serum albumin (BSA) (Sigma-Aldrich, Steinheim, Germany) in DPBS buffer (Gibco, Scotland, UK) for 60 minutes at 37°C. Before adding MCF-7 and MDA-MB-231 cells into the cell adhesion assay, cancer cells were stained with Calcein-AM (BD Pharmingen, San Jose, CA, USA), and tested inhibitors (C-3353, C-3380, C-3389, C-3399) were added at the concentrations of 3, 10, 30, 50 and 100 µM 15 min before adding MCF-7 and MDA-MB-231 to the adhesive surface. Then, cancer cells were left to adhere to collagen I for 30 min at 37°C. Non-adherent cells were gently washed twice with DPBS, and attached cells were counted in nine randomly selected visual fields for each well using a CQ1 image cytometer (Yokogawa, Tokyo, Japan). Experiments were conducted twice in six technical replicates. The images were analyzed using Columbus 2.4.2 software (Perkin Elmer, Waltham, MA, USA). The mean inhibition of adhesion for visual fields was calculated by using the equation: % of control = [number of adhered cells in treated samples/number of adhered cells in control group] ×100%.

**Electric cell-substrate impedance-sensing assays (ECIS)**

The migration of MCF-7 and MDA-MB-231 cells was monitored using real-time quantitative wound healing assays and 96W1E+ ECIS arrays (Applied Biophysics, Troy, NY, USA) according to the procedure reported by Stojak et al. (2020) with minor modifications. To this end, MDA-MB-231 cells were seeded at a density of 3 × 10^4^ per well and MCF-7 cells at a density of 6 × 10^4^ cells per well. Before the cell seeding procedure, the 96W1E+ plate was pretreated with 10 mM L-cysteine (Sigma-Aldrich, Steinheim, Germany) according to the manufacturer’s instruction. Immediately after cell seeding, resistance (Ω), capacitance (µF) and impedance (Ω) measurements were initiated at frequencies from 250 Hz to 64 kHz (250, 500, 1000, 2000, 4000, 8000, 16,000, 32,000 and 64,000 Hz) using the multiple frequency time mode. The cell-free wells served as a negative control to provide the baseline changes in impedance for all experiments. Wounding was performed by applying an alternating current of 3000 µA, 40 kHz for 30 s, killing the cells on the surface of the electrode, which resulted in an abrupt decrease in impedance to values similar to those of a cell-free electrode. The dead cells were washed away, and fresh medium was added or medium containing C-3353 inhibitor at various concentrations (10, 30 or 100 µM). The healing process was measured continuously as cells migrated onto the electrode. The area under the curve (AUC) was quantified and normalized to the untreated control. The experiment was performed in a humidified 5% CO_2_ incubator at 37 °C and was repeated twice in seven technical replicates.

1. Boschetti E, Righetti PG. Detailed methodologies and protocols. In: Low-abundance proteome discovery: state of the art and protocols. Elsevier, 2013.
2. Kalvins I, Chlopicki S, Andrianov V, Stojak M, Domraceva I, Kanepe-Lapsa I et al (2021) Aromatic sulphonamides derivatives that inhibits PDIA1, their synthesis and use. Patent no.: WO/2021/141506.
3. Chlopicki S, Kalvins I, Przyborowski K, Stojak M, Andrianov V, Domraceva I et al. (2021) Aromatic sulphonamides derivatives that inhibits PDIA3, their synthesis and use. Patent no.: WO/2021/141507.
4. Sitek B, Waldera-Lupa DM, Poschmann G, Meyer HE, Stühler K. Application of label-free proteomics for differential analysis of lung carcinoma cell line A549. Methods Mol Biol. 2012;893:241-248.
5. Kurpińska A, Suraj J, Bonar E, Zakrzewska A, Stojak M, Sternak M et al. Proteomic characterization of early lung response to breast cancer metastasis in mice. Exp Mol Pathol. 2019;107:129-140.
6. Ishihama Y, Oda Y, Tabata T, Sato T, Nagasu T, Rappsilber J et al. Exponentially modified protein abundance index (emPAI) for estimation of absolute protein amount in proteomics by the number of sequenced peptides per protein. Mol Cell Proteomics. 2005;4:1265-1272.
7. Roy J, Wycislo KL, Pondenis H, Fan TM, Das A. Comparative proteomic investigation of metastatic and non-metastatic osteosarcoma cells of human and canine origin. PLoS One. 2017;12(9):e0183930.
8. Stojak M, Milczarek M, Kurpinska A, Suraj-Prazmowska J, Kaczara P, Wojnar-Lason K et al. Protein disulphide isomerase A1 is involved in the regulation of breast cancer cell adhesion and transmigration via lung microvascular endothelial cells. Cancers. 2020;12(10):2850.
9. Malinowska A, Kistowski M, Bakun M, Rubel T, Tkaczyk M, Mierzejewska J et al. Diffprot - software for non-parametric statistical analysis of differential proteomics data. J Proteomics. 2012;75,4062-4073.
10. Silva JC, Gorenstein MV, Li G-Z, Vissers JPC, Geromanos SJ. Absolute quantification of proteins by LCMS : a virtue of parallel ms acquisition. MCP. 2006;5(1):144-156.
11. Psurski M, Janczewski Ł, Świtalska M, Gajda A, Goszczyński TM, Oleksyszyn J et al. Novel phosphonate analogs of sulforaphane: Synthesis, in vitro and in vivo anticancer activity. Eur J Med Chem. 2017;132:63-80.
