# Supplementary material for: Comparison of anti-cancer effects of novel protein disulphide isomerase (PDI) inhibitors in breast cancer cells characterized by high and low PDIA17 expression
Source: Cancer Cell Int. 2022 Jun 20;22:218. doi: 10.1186/s12935-022-02631-w (PMC9208212; doi:10.1186/s12935-022-02631-w)
Supplement: Supplementary file 2 — Additional file 2: Table S1. The origin and conditions of maintenance of cell cultures. [file 12935_2022_2631_MOESM2_ESM.docx]

**Comparison of anti-cancer effects of novel protein disulphide isomerase (PDI) inhibitors in breast cancer cells characterized by high and low PDIA17 expression**

Kurpińska Anna^1^, Suraj-Prażmowska Joanna^1^, Stojak Marta^1^, Jarosz Joanna^2^, Mateuszuk Łukasz^1^, Niedzielska-Andres Ewa^3^, Smolik Magdalena^3^, Wietrzyk Joanna^2^, Kalvins Ivars^4*^, Walczak Maria^1,3*^, Chłopicki Stefan^1,5*^

^1^ Jagiellonian University, Jagiellonian Centre for Experimental Therapeutics (JCET), Bobrzynskiego 14, 30-348, Krakow, Poland

^2^ Hirszfeld Institute of Immunology and Experimental Therapy, Department of Experimental Oncology, Polish Academy of Sciences, Rudolfa Weigla 12, 53-114, Wroclaw, Poland.

^3^ Jagiellonian University Medical College, Faculty of Pharmacy, Chair and Department of Toxicology, Medyczna 9, 30-688, Krakow, Poland

^4^ Latvian Institute of Organic Synthesis, Laboratory of Carbofunctional Compounds, LV-1006, Riga, Latvia

^5^ Jagiellonian University Medical College, Faculty of Medicine, Chair of Pharmacology, Grzegorzecka 16, 31-531, Krakow, Poland

*Corresponding authors: Stefan Chlopicki, stefan.chlopicki@jcet.eu, Maria Walczak, maria.walczak@jcet.eu, Ivars Kalvins, ivars.kalvins@lza.lv

**Additional file 2: Table S1**. The origin and conditions of maintenance of cell cultures.

| **No.** | **Cell line** | **Type of cancer** | **Purchased from** | **Medium** | **Number of passages used** |
| --- | --- | --- | --- | --- | --- |
| 1 | LNCaP | Prostate cancer | American Type Culture Collection (ATCC, Manassas, USA) | RPMI1640 GlutaMAX + Opti-MEM GlutaMAX medium (1:1) with 10% FBS, 3.5 g/L glucose, and  0,5 mM sodium pyruvate, AAS (100 units/ml of penicillin, 0,1 mg/ml streptomycin) | 3 |
| 2 | PC-3 |  | American Type Culture Collection (ATCC, Manassas, USA) | RPMI1640 medium with 10% FBS, 2 mM L-glutamine AAS (100 units/ml of penicillin, 0,1 mg/ml streptomycin) | 5 |
| 3 | Du-145 |  | American Type Culture Collection (ATCC, Manassas, USA) | Eagle’s medium, with 10% FBS, 1mM sodium pyruvate, 4 mM L-glutamine, AAS (100 units/ml of penicillin, 0,1 mg/ml streptomycin) | 3 |
| 4 | TRAMP C-2 |  | American Type Culture Collection (ATCC, Manassas, USA) | DMEM medium with 5% FBS, 4 mM L-glutamine, insulin (5µg/ml), trans-dehydroandrosteron, 5% NuSerum Culture Supplement, AAS (100 units/ml of penicillin, 0,1 mg/ml streptomycin) | 3 |
| 5 | TRAMP C-1 |  | American Type Culture Collection (ATCC, Manassas, USA) |  | 3 |
| 6 | Lovo | Colon cancer | American Type Culture Collection (ATCC, Manassas, USA) | RPMI1640 + Opti-MEM medium (1:1) with 5% FBS, 1mM sodium pyruvate, 2 mM L-glutamine, AAS (100 units/ml of penicillin, 0,1 mg/ml streptomycin) | 3 |
| 7 | HT29 |  | American Type Culture Collection (ATCC, Manassas, USA) | RPMI1640 + Opti-MEM medium (1:1), with 5% FBS, 1mM sodium pyruvate, 2 mM L-glutamine AAS (100 units/ml of penicillin, 0,1 mg/ml streptomycin and) | 3 |
| 8 | CaCO2 |  | Received as a gift from prof. Ivars Kalvins, Latvian Institute of Organic Synthesis, Riga, Latvia | Eagle’s medium, with 20% FBS, 1%  MEM non-essential amino acid solution, 1mM sodium pyruvate, 2 mM L-glutamine and AAS (100 units/ml of penicillin, 0,1 mg/ml streptomycin) | 3 |
| 9 | 5637 |  | Riken BRC Cell Bank - Japan | RPMI1640 GlutaMAX with 10% FBS, AAS (20 units of penicillin, 20 mg streptomycin) | 3 |
| 10 | MDA MB 231 | Breast cancer | American Type Culture Collection (ATCC, Manassas, USA) | RPMI1640 medium with 10% FBS, 2 mM L-glutamine, AAS (100 units/ml of penicillin, 0,1 mg/ml streptomycin) | 3 |
| 11 | 67NR |  | Barbara Ann Karmanos Cancer Institute, Detroit, Michigan, USA | DMEM medium with 10% CBS (calf bovine serum), 1% MEM non-essential amino acids solution, 2 mM L-glutamine, AAS (100 units/ml of penicillin, 0,1 mg/ml streptomycin ) | 3 |
| 12 | MCF-7 |  | American Type Culture Collection (ATCC, Manassas, USA) | Eagle’s medium, with 10% FBS, 1%  MEM non-essential amino acid solution, 0.8 mg/L insulin,  2 mM L-glutamine, AAS (100 units/ml of penicillin, 0,1 mg/ml streptomycin) | 5 |
| 13 | 4T1 |  | American Type Culture Collection (ATCC, Manassas, USA) | RPMI1640 GlutaMAX + Opti-MEM medium (1:1) with 10% FBS, 1mM sodium pyruvate, 3.5 g/L glucose AAS (100 units/ml of penicillin, 0,1 mg/ml streptomycin ) | 3 |
| 13 | T47D |  | American Type Culture Collection (ATCC, Manassas, USA) | RPMI1640 GlutaMAX medium with 5% FBS, 2 mM L-glutamine ,8 µg/mL insulin, AAS (100 units/ml of penicillin, 0,1 mg/ml streptomycin) | 3 |
| 15 | LLC | Lung cancer | Received as a gift from dr I. Wodinsky, National Cancer Institute, Bethesda, USA | DMEM medium with 10% FBS, 4 mM L-glutamine, 3.5 g/L glucose, AAS (100 units/ml of penicillin, 0,1 mg/ml streptomycin ) | 2 |
| 16 | NCI H1703 |  | American Type Culture Collection (ATCC, Manassas, USA) | RPMI1640 GlutaMAX with 10% FBS, AAS (20 units of penicillin, 20 mg streptomycin) | 2 |
| 17 | A549 |  | American Type Culture Collection (ATCC, Manassas, USA) | RPMI1640 + Opti-MEM medium (1:1), with 5% FBS, 2 mM L-glutamine AAS (100 units/ml of penicillin, 0,1 mg/ml streptomycin) | 2 |
| 18 | A427 |  | American Type Culture Collection (ATCC, Manassas, USA) | Eagle’s medium, with 10% FBS, 1%  MEM non-essential amino acid solution,  2 mM L-glutamine, 1 mM sodium puryvate, AAS (100 units/ml of penicillin, 0,1 mg/ml streptomycin) | 2 |
| 19 | NCI-H358 |  | Received as a gift from prof. Zdzisław Krawczyk, Maria Sklodowska-Curie Institute – Oncology Centre (MSCI), branch in Gliwice | RPMI1640 GlutaMAX with 10% FBS, AAS (100 units/ml of penicillin, 0,1 mg/ml streptomycin) | 3 |
| 20 | NCI H1299 |  | Received as a gift from prof. Zdzisław Krawczyk, Maria Sklodowska-Curie Institute – Oncology Centre (MSCI), branch in Gliwice | RPMI1640 GlutaMAX with 10% FBS, 2.5 g/L glucose, 1 mM sodium puryvate, AAS (100 units/ml of penicillin, 0,1 mg/ml streptomycin) | 2 |
| 21 | A2780 | Ovarian cancer | European Collection of Authenticated Cell Cultures (ECACC, Porton Down, England) | RPMI1640 GlutaMAX with 10% FBS, AAS (100 units/ml of penicillin, 0,1 mg/ml streptomycin) | 2 |
| 22 | HT1080 | Fibrosarcoma | Received as a gift from prof. Ivars Kalvins, Latvian Institute of Organic Synthesis, Riga, Latvia | DMEM-GlutMAX medium with 10% FBS and AAS (20 units of penicillin, 20 mg streptomycin) | 3 |
| *23** | *BALB/3T3 clone A31* | *Normal mouse fibroblasts cell line* | *American Type Culture Collection (ATCC, Manassas, USA)* | *DMEM medium with 10% FBS, 2 mM L-glutamine, AAS (100 units/ml of penicillin, 0,1 mg/ml streptomycin)* | **for IC50 evaluation-testing the toxicity of the compounds* |
| *24#* | *MCF-10A* | *normal human breast epithelial cell line* | *American Type Culture Collection (ATCC, Manassas, USA* | *Ham’s F12 with 5% HS, 10 mM non-essential amino acids, 2 mM L-glutamine, 500 ng/ml hydrocortisone, 20 ng/ml human recombinant epidermal growth factor (EGF), 1 mg/ml insulin, 10 mg/ml bovine pituitary extract, AAS (100 units/ml of penicillin, 0,1 mg/ml streptomycin)* | *# for IC50 evaluation-testing the toxicity of the compounds* |
