# Supplementary material for: Comparison of anti-cancer effects of novel protein disulphide isomerase (PDI) inhibitors in breast cancer cells characterized by high and low PDIA17 expression
Source: Cancer Cell Int. 2022 Jun 20;22:218. doi: 10.1186/s12935-022-02631-w (PMC9208212; doi:10.1186/s12935-022-02631-w)
Supplement: Supplementary file 3 — Additional file 3: Table S2. The half maximal inhibitory concentration for selected PDIA1, PDIA3 and PDIA17 inhibitors. [file 12935_2022_2631_MOESM3_ESM.docx]

**Comparison of anti-cancer effects of novel protein disulphide isomerase (PDI) inhibitors in breast cancer cells characterized by high and low PDIA17 expression**

Kurpińska Anna^1^, Suraj-Prażmowska Joanna^1^, Stojak Marta^1^, Jarosz Joanna^2^, Mateuszuk Łukasz^1^, Niedzielska-Andres Ewa^3^, Smolik Magdalena^3^, Wietrzyk Joanna^2^, Kalvins Ivars^4*^, Walczak Maria^1,3*^, Chłopicki Stefan^1,5*^

^1^ Jagiellonian University, Jagiellonian Centre for Experimental Therapeutics (JCET), Bobrzynskiego 14, 30-348, Krakow, Poland

^2^ Hirszfeld Institute of Immunology and Experimental Therapy, Department of Experimental Oncology, Polish Academy of Sciences, Rudolfa Weigla 12, 53-114, Wroclaw, Poland.

^3^ Jagiellonian University Medical College, Faculty of Pharmacy, Chair and Department of Toxicology, Medyczna 9, 30-688, Krakow, Poland

^4^ Latvian Institute of Organic Synthesis, Laboratory of Carbofunctional Compounds, LV-1006, Riga, Latvia

^5^ Jagiellonian University Medical College, Faculty of Medicine, Chair of Pharmacology, Grzegorzecka 16, 31-531, Krakow, Poland

*Corresponding authors: Stefan Chlopicki, stefan.chlopicki@jcet.eu, Maria Walczak, maria.walczak@jcet.eu, Ivars Kalvins, ivars.kalvins@lza.lv

**Additional file 3: Table S2**. The half maximal inhibitory concentration for selected PDIA1, PDIA3 and PDIA17 inhibitors.

| **Inhibitor** | **IC50**  **[µM]** | | | **Structure of the inhibitors** |
| --- | --- | --- | --- | --- |
|  | **PDIA1** | **PDIA3** | **PDIA17** |  |
| **C-3380** | 0.033 | 37 | n.a. | 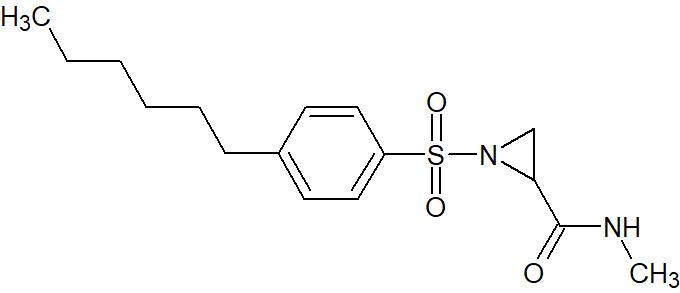 |
| **C-3389** | 0.03 | 23.3 | n.a. | 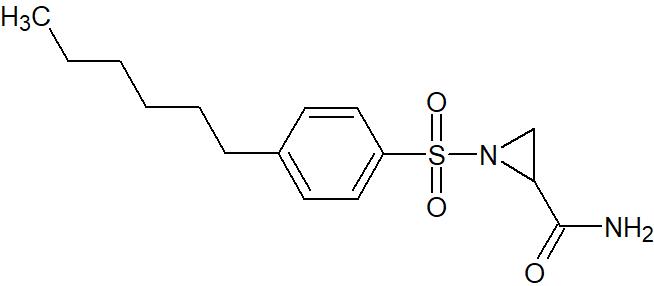 |
| **C-3399** | 0.12 | 4 | >200 | 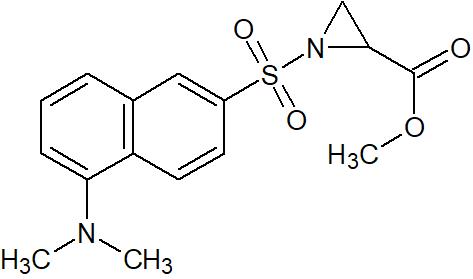 |
| **C-3353** | 5 | 143 | 20.1 | 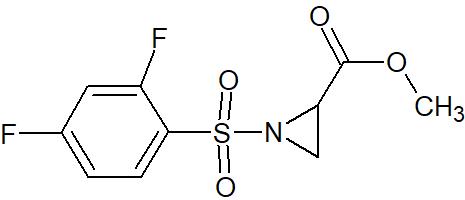 |

n.a. – not active in range of concentrations used
